# “Keeping an eye on amylase”. Side effects of antidepressants

**DOI:** 10.1192/j.eurpsy.2023.1760

**Published:** 2023-07-19

**Authors:** T. Jiménez Aparicio, G. Medina Ojeda, A. Rodríguez Campos, L. Rodríguez Andrés, C. Vallecillo Adame, C. De Andrés Lobo, M. Queipo de Llano de la Viuda, G. Guerra Valera, A. A. Gonzaga Ramírez, M. J. Mateos Sexmero, M. Fernández Lozano, B. Rodríguez Rodríguez, N. Navarro Barriga, M. P. Pando Fernández, P. Martínez Gimeno, M. Calvo Valcárcel, M. A. Andreo Vidal

**Affiliations:** Servicio de Psiquiatría, Sacyl, Hospital Clínico Universitario Valladolid, Valladolid, Spain

## Abstract

**Introduction:**

Both in consultations with the general practitioner and with the psychiatrist, antidepressants are one of the most used drugs (1). These have multiple indications, and there are different groups according to their mechanism of action. In relation to this case, we are going to talk about Venlafaxine, a dual-type antidepressant, that is, it inhibits the reuptake of serotonin and norepinephrine. One of the most common side effects is digestive discomfort, which usually resolves after a few weeks (2). However, we should not ignore these symptoms, since they can hide something more serious.

**Objectives:**

Presentation of a clinical case on a patient who presented an increase in pancreatic amylase after starting treatment with Venlafaxine.

**Methods:**

Bibliographic review including the latest articles in Pubmed on side effects of antidepressant treatment, and more specifically at the gastrointestinal level (in this case we will talk about pancreatitis).

**Results:**

We present the case of a 49-year-old woman, who was hospitalized 2 years ago, due to a first depressive episode. During this admission, psychopharmacological treatment was started for the first time, on that occasion with a selective serotonin reuptake inhibitor (SSRI), treatment of first choice (3). The patient had no side effects at that time, but the response was very modest, so it was decided to replace that antidepressant with Venlafaxine (with dual action), up to 150mg. The depressive symptoms improved markedly, however the patient began to feel digestive discomfort (which at first did not seem to be of great importance). A general analysis was performed, in which an increase in lipase (978 U/L) and amylase (528 U/L) was detected. An echoendoscopy, an abdominal scan, and a magnetic resonance cholangiography were performed; Pancreatitis secondary to drugs was suspected (a severe condition). Luckily, no significant lesions were found in the tests, and the levels of amylase and lipase decreased when Venlafaxine treatment was withdrawn (without reaching the normal range). The patient was discharged and continued to attend consultations. In the last control, amylase had dropped to 225 U/L. His abdominal pain disappeared. Treatment with Vortioxetine (a multimodal antidepressant) was started, however the amylase levels continue to be monitored, and the patient continues to see the gastroenterologist.

**Conclusions:**

Gastrointestinal side effects are very common when taking antidepressant treatment, and in most cases they do not usually represent a serious problem.

However, it is described in the scientific literature that in some cases, acute pancreatitis secondary to some drugs, including Venlafaxine, can occur (4). In order to detect it, it is necessary to perform a blood test and sometimes also other complementary tests.

For its treatment, the fundamental thing is to withdraw the causing drug, trying to find other alternatives, and carry out a control to monitor possible complications

**Disclosure of Interest:**

None Declared

